# Connectomic disturbances underlying insomnia disorder and predictors of treatment response

**DOI:** 10.3389/fnhum.2022.960350

**Published:** 2022-08-10

**Authors:** Qian Lu, Wentong Zhang, Hailang Yan, Negar Mansouri, Onur Tanglay, Karol Osipowicz, Angus W. Joyce, Isabella M. Young, Xia Zhang, Stephane Doyen, Michael E. Sughrue, Chuan He

**Affiliations:** ^1^Department of Rehabilitation Medicine, The Affiliated Jiangsu Shengze Hospital of Nanjing Medical University, Suzhou, China; ^2^Department of Radiology, The Affiliated Jiangsu Shengze Hospital of Nanjing Medical University, Suzhou, China; ^3^Omniscient Neurotechnology, Sydney, NSW, Australia; ^4^International Joint Research Center on Precision Brain Medicine, XD Group Hospital, Xi’an, China; ^5^Shenzhen Xijia Medical Technology Company, Shenzhen, China

**Keywords:** Insomnia, machine learning, functional connectivity, rTMS, treatment response

## Abstract

**Objective:**

Despite its prevalence, insomnia disorder (ID) remains poorly understood. In this study, we used machine learning to analyze the functional connectivity (FC) disturbances underlying ID, and identify potential predictors of treatment response through recurrent transcranial magnetic stimulation (rTMS) and pharmacotherapy.

**Materials and methods:**

51 adult patients with chronic insomnia and 42 healthy age and education matched controls underwent baseline anatomical T1 magnetic resonance imaging (MRI), resting-stage functional MRI (rsfMRI), and diffusion weighted imaging (DWI). Imaging was repeated for 24 ID patients following four weeks of treatment with pharmacotherapy, with or without rTMS. A recently developed machine learning technique, Hollow Tree Super (HoTS) was used to classify subjects into ID and control groups based on their FC, and derive network and parcel-based FC features contributing to each model. The number of FC anomalies within each network was also compared between responders and non-responders using median absolute deviation at baseline and follow-up.

**Results:**

Subjects were classified into ID and control with an area under the receiver operating characteristic curve (AUC-ROC) of 0.828. Baseline FC anomaly counts were higher in responders than non-responders. Response as measured by the Insomnia Severity Index (ISI) was associated with a decrease in anomaly counts across all networks, while all networks showed an increase in anomaly counts when response was measured using the Pittsburgh Sleep Quality Index. Overall, responders also showed greater change in all networks, with the Default Mode Network demonstrating the greatest change.

**Conclusion:**

Machine learning analysis into the functional connectome in ID may provide useful insight into diagnostic and therapeutic targets.

## Introduction

Insomnia disorder (ID) is the most common form of sleep disorder, characterized by difficulties in initiating and maintaining sleep, along with early morning waking (Morin et al., 2015). Additionally, ID is associated with mood disorders (e.g., anxiety, depression) and neurodegenerative disorders (e.g., Parkinson’s disease and dementia). Even if sufferers experience insomnia alone, they have an increased risk of developing both physical and mental health problems (Taylor et al., 2021). Furthermore, compared to good sleepers, ID sufferers experience poor daytime performance and poor cognitive function, with subsequent impairment in quality of life and well-being (Fortier-Brochu et al., 2012). However, diagnosing ID can be a complex task given the heterogeneity and lack of specificity of symptoms; thus, more sophisticated techniques are required to establish methods for assessing ID.

Early detection and management of ID remains a clinical priority and is necessary to mitigate the effects and associated risks of ID. The gold standard for evaluating ID is polysomnography (PSG), however, PSG data do not reflect the sleep problems reported by approximately 40% of ID patients (Krystal et al., 2002). Moreover, PSG is not used regularly due to the need for a specialized setting and equipment, and how labor intensive it is. Wrist actigraphy provides similar insights to PSG, but lacks specificity (Hyde et al., 2007; Morgenthaler et al., 2007; Gruwez et al., 2017), minimizing its utility in ID assessment. Due to the limitations of established objective measures, self-reported sleep instruments remain the most practical methods for ID assessment in clinical practice (Buysse et al., 2010; Van de Water et al., 2011). Conversely, while the clinical utility of self-reported sleep instruments is axiomatic, these measurements fail to provide objective markers of ID and do not provide any mechanistic insights or biological targets.

More recently, functional connectivity (FC) derived from resting state functional magnetic resonance imaging (rsfMRI) has been used to characterize changes in ID (Pang et al., 2017; Yan et al., 2018; Fasiello et al., 2022). Functional connectivity analysis provides an accessible and efficient method for diagnosis, especially given the large-scale network changes observed in ID. ID sufferers demonstrate aberrant functional connectivity across several networks, including the Default Mode Network (Pang et al., 2017; Li et al., 2018; Ma et al., 2018; Zhou et al., 2018; Fasiello et al., 2022), Salience Network (Liu et al., 2018; Wei et al., 2020; Fasiello et al., 2022), Central Executive Network (Liu et al., 2018; Zhou et al., 2018; Wei et al., 2020; Fasiello et al., 2022), and Sensorimotor Network (Dai et al., 2020; Fasiello et al., 2022). However, the significance of these connectomic differences in ID is not entirely understood, with inconsistencies in networks and directions of changes across studies, and discordance between structural and functional changes (Tahmasian et al., 2018).

Given the complexity and scale of both ID and FC, machine learning approaches may assist in better understanding network changes in ID, and evaluating whether FC has the potential to act as a diagnostic tool. While machine learning tools have been used to identify sleep stages from FC data (Tagliazucchi et al., 2012; Tagliazucchi and Laufs, 2014), only one previous study has used machine learning to distinguish ID from controls. In their work, He et al. utilized simultaneous EEG-fMRI to record multiple fMRI sessions during a specific sleep stage, to which they applied a support vector machine for classification (He et al., 2022). While their model had high accuracy, it was unable to provide the improved accessibility which would result from the ability to perform machine learning on a single awake fMRI for diagnosis. In addition, they were unable to perform feature importance to identify which features of the machine learning model, or areas of the brain, were contributing most to the model’s classification. The identification of these brain network features is crucial, as they may provide (1) biomarkers used to diagnose ID, (2) insight into the pathogenesis of ID, and (3) aid in identification of brain modulation targets for treatment.

Given its prevalence and burden, several treatment strategies have been investigated to improve sleep in ID. Cognitive behavioral therapy (CBT) is often recommended as first-line treatment; however, it is often underutilized due to barriers around access to trained providers, time burden, or economic issues (Rossman, 2019). Given the limitations of CBT, pharmacotherapy remains a common approach, which is both effective and accessible, though is associated with potential adverse effects and dependence (Lie et al., 2015), limiting its utility. Further understanding of the neural mechanisms underlying ID may enable utilization of newer techniques, such as repetitive transcranial magnetic stimulation (rTMS) to complement existing therapeutic approaches.

Repetitive transcranial magnetic stimulation (rTMS) has been suggested as a candidate therapy for reducing cortical excitability and hyperarousal associated with ID, and in the long-term, modulating plasticity of neuronal networks which may promote the release of sleep-related hormones (van der Werf et al., 2010; Lanza et al., 2015; Feng et al., 2019). Several studies have demonstrated improved sleep quality following rTMS in ID, with a recent meta-analysis demonstrating superiority to sham in improving several sleep-related measures, including sleep efficiency, sleep time, and number of awakenings (Ma et al., 2021). Despite its promise, research in other disorders (such as major depressive disorder) suggests that rTMS demonstrates substantial variability in treatment efficacy across individuals (Kar, 2019). Therefore, markers of predicting rTMS response are key to identifying suitable cohorts who may benefit from rTMS treatment of ID.

In this study, we applied a recently described machine learning approach, Hollow Tree Super (HoTS) (Doyen et al., 2021), specifically designed to handle high-dimensional functional connectivity fMRI data, to a clinical cohort of ID in order to investigate two clinically relevant questions. (1) Can a single awake rsfMRI scan differentiate subjects with ID from controls; testing the potential utility of fMRI in diagnosing ID. (2) How do large-scale brain networks respond to combined pharmacotherapy and rTMS; attempting to identify rTMS response markers and new potential stimulation targets.

## Materials and methods

### Study design

The study was an open-label, prospective, randomized study investigating the functional connectivity disturbances underlying ID, and how large-scale networks respond to pharmacotherapy and rTMS among healthy volunteers and ID patients. We enrolled 51 right-handed older adult patients with ID and 42 healthy older adult volunteers who were age and education matched but did not have insomnia (recruited from Jiangsu, China, from July 2020 to July 2021). A simple randomization was performed to randomly allocate 24 of the 51 ID patients to drug only or combined pharmacotherapy and TMS treatment groups. The study was approved by the hospital’s Human Research Ethics Committee and registered with the Chinese Clinical Trials Registry (ChiCTR2100049455). All participants provided written informed consent according to the Declaration of Helsinki prior to data collection. The clinical activities were open label to both participants and physicians, whereas the analysis was performed by blinded authors on de-identified data. *A priori* sample size estimation was performed using G Power version 3.1, according to previous research (Jiang et al., 2019). Healthy age-matched adult volunteers had a questionnaire assessment (see *Assesment* section for details) and MRI [resting-state functional MRI (rsfMRI), T1-weighted imaging, and diffusion weighted imaging (DWI) (see *Imaging Protocol* section for details)] at baseline. ID patients underwent questionnaire assessment and MRI scan at baseline and four weeks following treatment. All questionnaires were carried out prior to the MRI scan. To classify patients with ID from healthy controls based on FC, we used a machine learning method to fit the model, which was evaluated with the mean area under the receiver operating characteristic curve (AUC-ROC). The treatment response was dependent on the Pittsburgh Sleep Quality Index (PSQI) and the Insomnia Severity Index (ISI) measured before and after treatment. (see Assessment for specific instructions).

### Patient cohort

Fifty-one patients with Insomnia Disorder were recruited from the Outpatient Department of Rehabilitation of the Affiliated Jiangsu Shengze Hospital of Nanjing Medical University, while 46 healthy volunteers were recruited from the Wujiang Shengze Zhen Old-age University. Participants were aged 45–75 and had a junior high school education or above. All participants met the diagnostic criteria for ID in the Fifth Edition of the Diagnostic and Statistical Manual of Mental Disorders (American Psychiatric Association, 2013) and agreed to participate in the trial on a voluntary basis. Diagnostic interviews were conducted by two experienced neurologists. Patients were excluded based on the following criteria: concurrent psychotherapy or counseling and substance or alcohol abuse or dependence; evidence of neurological or other psychiatric diseases; pregnancy or breastfeeding women; any contraindication for rTMS (including implanted metal and devices in the body, and history of epilepsy).

Post treatment scans and assessments were collected for 24 participants with ID: 12 patients who received pharmacotherapy alone, and 12 patients who received both pharmacotherapy and rTMS. The median age (IQR) was 53.5 (8.0) for the pharmacotherapy group, and 57 (16.0) for the combined pharmacotherapy and rTMS group, while the median number of years of education was 9 for both groups.

### Repetitive transcranial magnetic stimulation protocol

The rTMS was performed using a MagPro device (MagPro X100, Tonica Elektronik A/SFarum, Denmark) connected with a figure-8-coil located above the right dorsolateral prefrontal cortex (rDLPFC) at a horizontal angle of 45^°^ relative to the midline of the nose. The rDLPFC was chosen as a target based on emerging literature suggesting this as an effective target for treating sleep difficulties in both disorders where sleep difficulties manifest as part of the syndrome [e.g., narcolepsy (Lanza et al., 2015)], and ID specifically. Magnetic pulses with a frequency of 1 Hz and an intensity of 90% of the motor threshold were delivered in 40 trains of 30s on and 8s off. Sessions were conducted five times per week for four weeks at 1200 pulses per session.

### Pharmacotherapy protocol

During the rTMS treatment period, both pharmacotherapy and combined rTMS and pharmacotherapy groups (24 ID participants) were given the following three phase pharmacotherapy protocol: the first phase (week 1) consisted of clonazepam (Enhua Pharma Co., Ltd, Xuzhou, China) 1 mg and zolpidem (Sanofi Pharma Co., Ltd, Hangzhou, China) 5 mg daily at bedtime; the second phase (weeks 2–3) consisted of clonazepam 1 mg and zolpidem 2.5 mg; while in the third phase (week 4) participants were administered only with clonazepam 1 mg. Throughout the study all ID patients were required to keep a daily sleep diary and follow the treatment prescription.

### Assessment

All assessments were completed by trained research assistants, who were blind to whether the patients were receiving rTMS combined with pharmacotherapy or pharmacotherapy only. The Alcohol Use Disorders Identification Test (AUDIT) and the Fagerstrom Test for Nicotine Dependence (FTND) were performed as baseline features for incorporation into the model to distinguish ID patients from healthy individuals. The Epworth Sleepiness Scale (ESS) was used to measure subjective daytime sleepiness and excessive daytime sleepiness (ESS > 10) (Arnulf et al., 2002). The Pittsburgh Sleep Quality Index (PSQI), an index of sleep quality; and the Insomnia Severity Index (ISI), an index of global insomnia severity; were evaluated to assess response to therapy.

### Imaging protocol

All subjects underwent an MRI scan on a 3.0T GE Discovery MR750w (SIGNA) scanner 24 channel head coil (Head 24). A 10-min, gradient-echo echo-planar imaging T2* sensitive pulse sequence was used to acquire resting-state fMRI data [interleaved sequence, slices = 41, thickness = 3.5 mm, pixel spacing = 3 × 3 mm, repetition time (TR) = 2500 ms, echo time (TE) = 30 ms, field of view (FOV) = 192 × 192 mm, flip angle = 90°, and acquisition matrix = 64 × 64, percent sampling = 100%, percent Phase Field of View = 100%].

A three-dimensional, 5-min, spoiled-gradient recalled T1-weighted sequence (axial 3D T1 BRAVO) was used to acquire whole-brain structural data with an acquisition time of 301s (slices = 188, thickness = 1 mm, pixel spacing = 1*1 mm, TR = 8.5 ms, TE = 3.2 ms, Inversion Time = 450 ms, Spacing Between Slice = 1, skip = 0 mm, flip angle = 12°, FOV = 256 × 256 mm, and acquisition matrix = 256 × 256, percent sampling = 100%, percent Phase Field of View = 100%).

Diffusion-weighted volume were acquired using the following parameters: 65 contiguous slices, slice thickness = 2.1 mm, FOV = 256 × 256 mm, matrix = 128 × 128 mm, TR = 17000 ms, TE = 95.9 ms, voxel size = 2 mm isotropic, acquisition number of excitations (NEX) = 1 partial Fourier, 64 diffusion directions with b-value = 1000 s/mm^2^, and 1 image with no diffusion weighting (*b* = 0 s/mm^2^), bandwidth = 250 Hz/pixel. Acquisition time was 19.16 min per diffusion-weighted scan.

### Diffusion tractography preprocessing steps

Diffusion weighted imaging (DWI) was processed using the Infinitome software (Omniscient Neurotechnology, 2020), which employs a standard processing steps in the Python language (Garyfallidis et al., 2014). This includes the following steps: (1) the diffusion image is resliced to ensure isotropic voxels, (2) motion correction is performed using a rigid body alignment, (2) slices with excess movement (defined as DVARS > 2 sigma from the mean slice) are eliminated, (3) the T1 image is skull stripped using a convolutional neural net (CNN). This is inverted and aligned to the DWI image using a rigid alignment, which is then used as a mask to skull strip the DWI image, (4) gradient distortion correction is performed using a diffeomorphic warping method which aims to locally similarize the DWI and T1 images, (5) eddy current correction is performed, (6) the fiber response function is estimated and the diffusion tensors are calculated using constrained spherical deconvolution, (7) deterministic tractography is performed with random seeding, usually creating about 300,000 streamlines per brain (Doyen et al., 2022).

### Creation of a personalized brain atlas using machine learning based parcelation

Since we sought to minimize the effects of gyral variation, we created a machine learning based, subject specific version of the Human Connectome Project Multimodal Parcelation (HCP-MMP1) atlas (Glasser et al., 2016) based on diffusion tractography structural connectivity, which we have described elsewhere (Doyen et al., 2022). In short, this was created by training a machine learning model on a separate cohort of 200 normal adult subjects by first processing T1 and DT images as above. An HCP-MMP1 atlas in NIFTI MNI space was then warped onto each brain and the structural connectivity was calculated between every pair of this atlas and a set of ROIs containing 8 subcortical structures per hemisphere as well as the brainstem based on the streamlines, which terminated within an ROI. This step allows the generation of feature vectors and generates a centroid of the parcelation, which is utilized to constrain the voxels studied in order to assign these to a given parcelation that is in a plausible area in the vicinity of its typical position. These feature vectors for each region were then used as a training set and the data were modeled using the XGBoost method (Chen and Guestrin, 2016).

This model was then applied to the new subject by first warping the HCP-MMP1 atlas to the new brain and collecting a set of feature vectors for the connectivity of each voxel. The feature vectors are then used to determine if each voxel belongs to a parcelation or region or not, and if so to assign the voxel to that parcelation. This creates a version of the HCP-MMP1 atlas with 180 cortical regions and 9 subcortical structures per hemisphere, along with the brainstem as one region, which is independent of brain shape or pathologic distortion, and that is specific for each subject.

The identified parcels were also automatically labeled by the Infinitome software to their known affiliations to large-scale networks, including the Accessory Language Network, Default Mode Network (DMN), the Central Executive Network (CEN), Dorsal Attention Network (DAN), Language Network, Limbic/Paralimbic Network, Salience Network (SN), Sensorimotor Network (SMN), and Visual Network (VN).

### Resting-stage functional magnetic resonance imaging preprocessing steps

The rsfMRI images were processed using standard processing steps which specifically include the following: (1) motion correction is performed on the T1 and BOLD images using a rigid body alignment, (2) slices with excess movement (defined as DVARS > 2 sigma from the mean slice) are eliminated, (3) the T1 image is skull stripped using a convolutional neural net (CNN), this is inverted and aligned to the resting state bold image using a rigid alignment, which is then used as a mask to skull strip the rsfMRI image, (4) slice time correction is performed, (5) global intensity normalization is performed, (6) gradient distortion correction is performed using a diffeomorphic warping method which aims to locally similarize the rsfMRI and T1 images, (7) high variance confounds are calculated using the CompCor method (Behzadi et al., 2007); these confounds as well as motion confounds are regressed out of the rsfMRI image, and the linear and quadratic signals are detrended. Note this method does not perform global signal regression, (8) spatial smoothing is performed using a 4 mm FWHM Gaussian kernel. The personalized atlas created in previous steps is registered to the T1 image and localized to the gray matter regions. Thus, it is ideally positioned for extracting an average BOLD time series from all 379 areas (180 parcelations × 2 hemispheres, plus 19 subcortical structures). This yields 143,641 correlations.

### Median absolute deviation outlier detection

In order to find outliers in functional connectivity in the ID group, we calculated the median and median absolute deviation (MAD) within the FC of the healthy controls. We then determined for each ID patient whether the FC was an outlier, based on a threshold of 3 or more MAD. The number of anomalies within each network were then counted and the mean calculated for responders and non-responders, before and after treatment. We also calculated the mean change in anomaly counts before and after treatment to compare responders to non-responders in the cohort of 24 patients who underwent treatment. This process was completed for responders on both the ISI and PSQI. For the ISI, response was defined as a change in the interpretive category (for example, improving from severe to moderate insomnia), whereas for the PSQI, the reliable change index (RCI) (Christensen and Mendoza, 1986) was used, and a statistically significant response was defined as an RCI of 1.96 or greater.

### Mapping of symptoms to brain regions using Hollow-Tree Super method

Machine learning was used to classify patients with ID from healthy controls based on the pairwise functional connectivity between the 379 regions of each individual’s brain atlas. We used an XGBClassifier, a boosted trees approach to fit the model, which provides a superior prediction ability than single trees. All models included age, sex, AUDIT score, and FTND score as features. We used 5-fold cross-validation, and evaluated the model with the mean area under the receiver operating characteristic curve (AUC-ROC).

The black box problem in machine learning poses a substantial limitation on the ability for machine learning models to identify which part of the brain a problem arises from, posing a barrier for clinical translation. To traverse this, we used a boosted trees approach, called HoTS, described elsewhere (Kar, 2019), to attempt to gain visibility over which features the model was using to classify subjects based on the input fMRI scans, with the obvious implication that these features were providing insight into what brain regions had abnormal connectivity that might be linked to the symptom or its response to treatment. The HoTS method is a useful approach when dealing with high-dimensional data (for instance functional connectomics) and can provide directional weights with interpretable magnitudes. This method has been custom designed for tree based models (which is used in the current study), built on the ELI5 method which linearizes a decision tree. In addition, the HoTs method is custom built for analyzing connectomic pairwise features, i.e., parcel-to-parcel connectivity. The extracted feature importances were visualized at a network-level, based on the importance of each brain network in the output of the model, and at an individual brain region level using a SHAP plot of the top 20 features contributing to the model. Each SHAP plot provides a list of features in descending order of importance, along with their impact on the model along the x-axis, with the color of each point indicating whether a high (red), or low (blue) value of that feature is associated with the model.

### Statistical analysis

A Chi-squared test of independence was used to compare nominal demographic variables between healthy controls and ID patients, whereas a Mann-Whitney U test was used to compare continuous variables. Furthermore, Fisher’s exact test was used to identify whether there was an association between treatment modality and outcome. All statistical analyses were performed in R version 4.1.0.

## Results

### Subject demographics

No study-related serious adverse events were identified. Table 1 shows the demographics and clinical characteristics of all subjects at baseline. The median age of healthy controls and ID patients was 56 years and 57 years, respectively. There was a significant difference in the ESS (*U* = 858.5, *p* = 0.022), ISI (*U* = 46.5, *p* < 0.001), and PSQI (*U* = 38, *p* < 0.001) between healthy controls and ID patients, with patients scoring significantly higher on each test.

**TABLE 1:** Baseline characteristics for the entire cohort.

Demographic	Healthy controls (*n* = 42)	Insomnia patients (*n* = 51)	*p*-value
Sex F/M (%)	35/7 (83.3/16.7)	37/14 (72.5/27.5)	0.225
Median age (IQR) *years*	56.0 (6.9)	57.0 (14.1)	0.375
Median AUDIT score (IQR)	0 (0)	0 (0)	0.869
Median FTND score (IQR)	0 (0)	0 (0)	0.908
Median ESS score (IQR)	2.0 (3.75)	4.0 (6.0)	0.022
Median ISI score (IQR)	0 (3.0)	15.0 (5.5)	< 0.001
Median PSQI score (IQR)	3.0 (3.75)	16.0 (4.0)	< 0.001

*AUDIT*, Alcohol Screening Tool; *FTND*, Fagerstrom Test for Nicotine Dependence; *ESS*, Epworth Sleepiness Scale; *ISI*, Insomnia Severity Index; *PSQI*, Pittsburgh Sleep Quality Index.

### Treatment outcomes following pharmacotherapy and repetitive transcranial magnetic stimulation

Table 2 demonstrates the demographics and clinical characteristics for the 24 subjects who underwent treatment with combined pharmacotherapy and rTMS, and pharmacotherapy alone. There was no statistically significant difference between the demographics of the two treatment groups. Insomnia patients who underwent both pharmacotherapy and rTMS demonstrated a greater decrease in median ESS, ISI, and PSQI scores at follow-up compared to patients who only received pharmacotherapy, though this difference was not statistically significant.

**TABLE 2:** Baseline and follow-up characteristics for insomnia patients who underwent treatment.

Demographic	Healthy controls (*n* = 42)	Insomnia patients (*n* = 24)	*P*-value	Insomnia patients drug only (*n* = 12)	Insomnia patients drug + rTMS (*n* = 12)	*P-value*
Sex F/M (%)	35/7 (83.3/16.7)	16/8 (66.7/33.3)	0.212	9/3 (75.0/25.0)	7/5 (58.3/41.7)	0.665
Median age (IQR) *years*	56.0 (6.9)	54.0 (14.25)	0.936	53.5 (8.0)	57.0 (16.0)	0.729
Median AUDIT score (IQR)	0 (0)	0 (0)	0.251	0 (0)	0 (0)	0.965
Median FTND score (IQR)	0 (0)	0 (0)	0.897	0 (0)	0 (0)	0.359
**Median ESS score (IQR)**						
Baseline	2.0 (3.75)	6.0 (7.0)	0.013	7.5 (6.5)	2.0 (7.0)	0.036
Follow-up	–	3.0 (5.0)		4.0 (3.5)	2.0 (3.75)	0.091
**Median ISI score (IQR)**						
Baseline	0 (3.0)	15.0 (7.25)	< 0.001	15.5 (7.75)	14.5 (4.5)	0.505
Follow-up	–	6.0 (6.0)		9.0 (4.25)	5.0 (3.25)	0.059
**Median PSQI score (IQR)**						
Baseline	3.0 (3.75)	15.5 (4.25)	< 0.001	17.0 (5.0)	15.0 (3.5)	0.641
Follow-up	–	10.5 (5.25)		13.0 (3.5)	8.0 (5.5)	0.076

*AUDIT*, Alcohol Screening Tool; *FTND*, Fagerstrom Test for Nicotine Dependence; *ESS*, Epworth Sleepiness Scale; *ISI*, Insomnia Severity Index; *PSQI*, Pittsburgh Sleep Quality Index; *rTMS*, repetitive Transcranial Magnetic Stimulation.

Next, defining improvement as either an RCI > 1.96 for the PSQI, and a decrease in severity category for the ISI, 22 subjects (91.7%) demonstrated improvement. 13 subjects (54.2%) showed improvement on both the PSQI and ISI. When improvement was measured using the PSQI, 10 subjects showed improvement if they received combined pharmacotherapy and rTMS (83.3%), whereas 8 subjects showed improvement if they received pharmacotherapy alone (66.7%). Fisher’s exact test did not reveal a significant association between treatment group and outcome (*p* = 0.640). When measuring improvement with the ISI, 9 (75.0%) subjects who received combined pharmacotherapy and rTMS showed improvement, whereas 8 (66.7%) subjects who only received pharmacotherapy showed improvement. Fisher’s exact test once again did not show a significant association between the treatment modality and outcomes (*p* = 1).

### Machine learning prediction of functional connectivity changes in insomnia disorder

**FIGURE 1:**
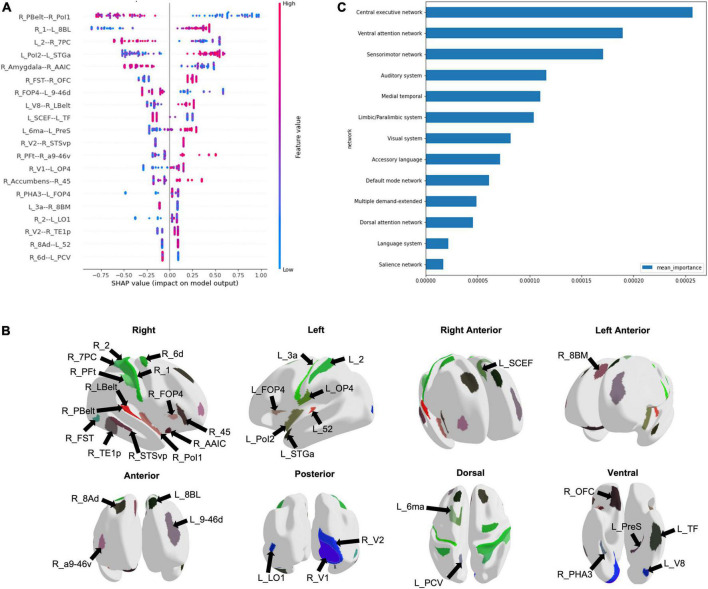
Machine learning modeling to classify subjects into Insomnia disorder and controls. **(A)** The functional connectivity among 39 brain regions comprised the top 20 features of the model, depicted here in a SHAP plot. The x-axis depicts the impact of each feature on the model output, while the color of each data point indicates whether a high or low functional connectivity is associated with the output. **(B)** The regions are demonstrated on a model brain. **(C)** The graph demonstrates the mean importance of each network on the model’s classification.

Using the entire cohort of 51 ID patients and 42 controls, machine learning was used to classify subjects into ID and control groups based on their FC. The best performance was achieved using an extreme gradient boosting model with hyperparameter tuning. This model included functional connectivity, sex, age, along with the AUDIT and FTND scores to classify subjects into ID or control. The mean AUC was 0.828. Using the HoTS method to extract feature importance revealed that the top 20 features were the functional connectivity among 39 brain regions (Figure 1A). These regions were mostly right sided, with many in the sensorimotor and insular regions (Figure 1B). Aggregating SHAP absolute values at a network level to identify the mean importance of each network revealed that the functional connectivity of regions within the CEN, VAN, and SMN had the greatest contribution to the model’s classification (Figure 1C).

### Responders demonstrate a greater number of anomalies in functional connectivity

Despite our initial aim, we were unable to predict ISI and PSQI improvement using machine learning, primarily due to large class imbalances. The subsequent analysis on improvement therefore solely utilized MAD outlier detection on the subgroup of 24 patients who underwent treatment, rather than HoTS-based machine learning approaches.

**FIGURE 2:**
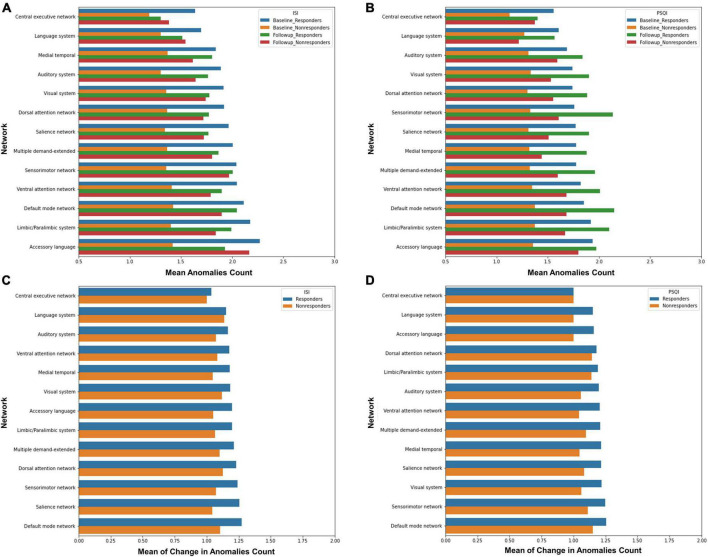
Median absolute deviation anomaly detection. Each graph depicts the mean anomaly count at baseline and follow-up for each network when response was measured using **(A)** the ISI, and, **(B)** the PSQI. The mean of the change in anomaly counts from baseline to follow-up in responders and non-responders have also been graphed for **(C)** the ISI, and, **(D)** the PSQI.

For the ISI, baseline anomaly counts were higher in responders compared to non-responders across all networks (Figure 2A). The accessory language network had the greatest number of anomalies in responders, followed by the Limbic/Paralimbic Network, DMN, and VAN. There was not a similarly hierarchical trend in anomaly counts in non-responders at baseline. Mean anomaly counts decreased at follow-up in responders in all networks, whereas the number of anomalies increased across all networks in non-responders. The accessory language network showed the greatest change following intervention, with the greatest decrease in anomalies in responders, and greatest increase in non-responders.

Similar to the ISI, for the PSQI response, baseline anomaly counts were higher in responders relative to non-responders across all networks (Figure 2B). Interestingly, however, almost all networks showed an increase in mean anomaly counts at the follow-up scan, in both responders and non-responders.

When looking at change in anomaly counts between responders and non-responders across the ISI and PSQI, responders demonstrated a greater change across all networks compared to non-responders (Figures 2C,D). For both the ISI and PSQI, the DMN showed the greatest change in anomalies. This was followed by the SN and SMN for the ISI, and SMN and VN for PSQI. However, it is important to note that this was a measure of absolute change, rather than a decrease or increase in anomalies.

**FIGURE 3:**
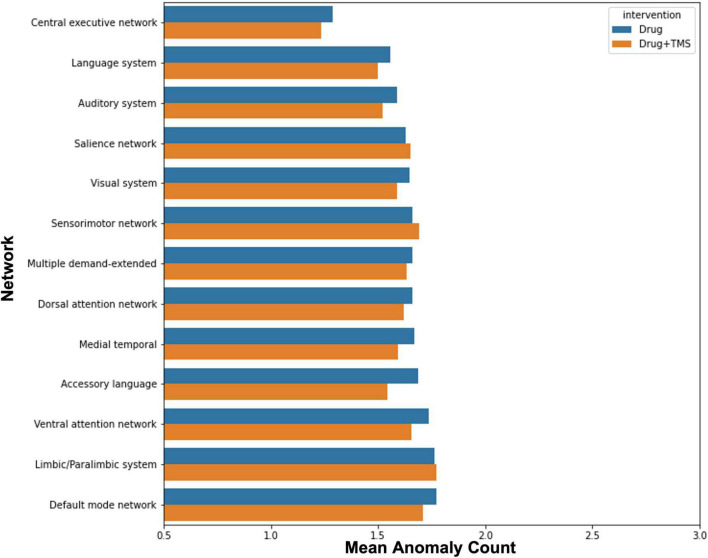
Effect of intervention on anomalies. The graph demonstrates the mean change in anomaly count by network for participants who received pharmacotherapy alone, and those who received pharmacotherapy and TMS.

Finally, we attempted to explore the effect of intervention (drug vs rTMS + drug) on anomalies in order to identify whether the addition of rTMS influenced anomaly count. There was no observable difference in change in anomaly count between intervention groups (Figure 3).

## Discussion

In this study, we performed functional connectivity-based analysis comparing ID patients with controls at baseline and post-intervention (pharmacotherapy or combined pharmacotherapy and rTMS) in order to identify diagnostic and therapeutic targets. Our machine learning model effectively classified ID, demonstrating that connectivity within the CEN, VAN, and SMN were most predictive of ID at baseline. Furthermore, exploration of connectome anomalies suggested that improvement following pharmacotherapy with or without rTMS was associated with a greater number of functional connectivity anomalies at baseline compared to non-responders. Overall, our results demonstrate that ID is a complex multi-network disorder which may benefit from machine learning and connectomic approaches to improve therapeutic options for patients. Our study is the first to utilize awake rsfMRI-based FC to differentiate ID from controls, and further extract features associated with classification, though further studies are necessary to validate our findings.

### Network changes associated with insomnia disorder

Our analysis highlighted several networks which contributed to the machine learning model’s classification of ID from controls, with the CEN demonstrating the greatest aggregated contribution. Several studies have shown alterations in the CEN FC in ID, though both increased and reduced connectivity have been reported (Fasiello et al., 2022). Although executive function testing has not been performed in the same studies to identify concordant changes, it is proposed that FC abnormalities in the CEN may be responsible for the daytime cognitive and emotional changes observed in ID (Liu et al., 2018; Zhou et al., 2018; Fasiello et al., 2022). Connectivity differences in the CEN may also support a reduced capacity to disengage from processing of external stimuli, which is consistent with the clinical characteristics of ID. ID patients have also demonstrated reduced functional connectivity between the anterior SN and left CEN (Wei et al., 2020). While the SN was not among the top networks contributing to our model’s classification, several regions out of the top 20 features, such as right Area Posterior Insular 1 (R_PoI1), right Frontal Opercular Area 4 (R_FOP4), and left Supplementary and Cingulate Eye Field (L_SCEF) are associated with the SN (Akiki and Abdallah, 2019). Given the proposed role of the SN in switching between the CEN and DMN (Goulden et al., 2014), these findings may be reflecting aberrancy in the ability to switch between networks in response to stimuli. While our analysis did not point to the DMN at baseline, several studies have inconsistently reported increased and decreased FC in the DMN in ID (Pang et al., 2017; Li et al., 2018; Ma et al., 2018; Zhou et al., 2018; Rossman, 2019). Despite the heterogeneous findings, the DMN’s role in sleep and introspection are likely associated with disturbed attentional processing in ID.

Our analysis also pointed to the VAN and SMN in predicting ID at baseline, echoing many of the connectivity changes observed in ID relating to alterations in networks associated with responding to external stimuli. The VAN has a role in orienting attention to external stimuli (Allan et al., 2020). While not well studied in ID, abnormal connectivity of the VAN perhaps contributes to shallow sleep and increased sensitivity to external stimuli. Furthermore, connectivity differences in the SMN in ID are well established in the literature. ID patients reveal increased FC in the visual pathway (Zhou et al., 2018; Fasiello et al., 2022), and increased FC connectivity within SMN networks has been associated with longer ID duration (Dai et al., 2020). Both the primary and secondary visual cortices, and sensorimotor regions were among the top 20 features predicting ID in our model. These findings may suggest that alterations in the SMN lead to abnormal sensory-perception processing with increased sensitivity to external stimulation and consequent hyperarousal.

Taken together, these primary network predictors of ID may indicate a disrupted sensitivity to, and control of processing environmental stimuli, leading to a sensory and motor hypervigilance, supporting the psychopathology of ID. However, the cross-sectional nature of the extant literature prevents the identification of a causative relationship between network changes and ID. Therefore, it is unclear whether connectivity changes cause ID, are caused by it, or are an incidental manifestation of ID. Further prospective studies are necessary to clarify the nature of connectomic disturbances in ID.

### Predicting treatment response in insomnia disorder

There have been several attempts to identify electroencephalographic, or polysomnographic predictive markers for treatment response in ID (Espie et al., 2001; Kalmbach et al., 2020; Sweetman et al., 2021). A reliable predictor of outcome across treatment modalities has not yet been found. Given that TMS has only recently been investigated in the context of ID, there have not been many attempts to identify predictors of response. One study utilizing EEG-based connectivity found that weaker connectivity between the region of the right dorsolateral prefrontal cortex and frontal, insular and limbic regions was associated with greater change in PSQI following rTMS over the right DLPFC (Shi et al., 2021). Despite a small sample size and a lack of a neuro-navigation system, this study parallels our finding that patients with more outliers in FC respond better to treatment. Based on both our findings and those reported by Shi et al. (2021), it could be speculated that normalization of anomalous FC may be the mechanism by which therapeutic response is achieved when using rTMS in ID. Interestingly, the accessory language network was associated with the greatest change in ISI in responders following rTMS. While the accessory language network has not been investigated in the context of ID, accessory language network regions are associated with multiple linguistic and cognitive/attentional control functions that play a role in self-referential and ruminative thoughts, error monitoring, and affective response (Hertrich et al., 2020), suggesting a heightened anomaly count within the network could be contributing to the nociferous patterns of thought supporting ID.

In our analysis, both responders and non-responders showed an increase in mean anomaly counts, especially in PSQI response. It may be suggested that sleep quality treatment response is solely dependent on baseline connectomics. Conversely, it may be that some network anomalies facilitate functional normalization through a compensatory change in network connectivity. The DMN showing the greatest change in total anomalies in responders may be indicative of this, as plasticity of the DMN may help compensate for deficits in other networks. Furthermore, given a greater degree of total change in anomalies across the ISI and PSQI was associated with treatment response, connectome plasticity overall, rather than normalization, may be more predictive of ID improvement. However, these interpretations are highly speculative and need to be tested in prospective research.

Though initially planned, we were unable to compare the efficacy of combined rTMS and pharmacotherapy to pharmacotherapy alone, and identify the impact of each modality on FC changes. Our results demonstrated that a greater percentage of those who received combined rTMS and pharmacotherapy improved, compared to those who received pharmacotherapy alone. This was, however, a marginal difference, and our study was underpowered to find any statistically significant associations between treatment modality and outcome. Similarly, while our anomaly analysis demonstrated no difference between each modality and changes in anomaly counts, our small sample size prohibits us from making conclusions about the influence of rTMS in ID. Larger trials are necessary to evaluate whether rTMS provides sufficient benefit in ID.

There are several limitations of the current study. As aforementioned, our small sample size limits the external validity of our findings. Due to the sample size, it was not possible to associate connectome changes with treatment (i.e., pharmacotherapy alone vs. combined pharmacotherapy and rTMS). Furthermore, the connectome abnormalities we have identified may not be purely associated with ID, but instead concomitant depression or anxiety. Therefore, larger prospective studies with more rigorous designs are required to validate our findings, and provide a basis for connectome based rTMS target identification and clinical translation.

## Conclusion

Our findings demonstrate the utility of resting state fMRI to explore the connectomic disturbances underlying Insomnia disorder and how networks respond to pharmacotherapy and rTMS. Specifically, we demonstrate that baseline disruptions in metacognitive and sensory networks are predictive of treatment response, suggesting those with more disrupted networks are better candidates for therapy, however, more research is necessary to validate these claims.

## Data availability statement

The original contributions presented in this study are included in the article/supplementary material, further inquiries can be directed to the corresponding authors.

## Ethics statement

The studies involving human participants were reviewed and approved by Ethics Committee of Jiangsu Shengze Hospital. The patients/participants provided their written informed consent to participate in this study.

## Author contributions

CH conceived and designed the study. QL, WZ, and HY performed the study and collected materials. NM wrote the code. NM and KO analyzed the results. KO and OT visualized the results. OT, IY, QL, and WZ wrote the manuscript. IY, MS, SD, and CH helped coordinate the study and reviewed the manuscript. All authors contributed to the article and approved the submitted version.

## References

[B1] Akiki T. J. Abdallah C. G. (2019). Determining the Hierarchical Architecture of the Human Brain Using Subject-Level Clustering of Functional Networks. *Sci. Rep.* 9:19290. 10.1038/s41598-019-55738-y 31848397 PMC6917755

[B2] Allan P. G. Briggs R. G. Conner A. K. O’Neal C. M. Bonney P. A. Maxwell B. D. (2020). Parcellation-based tractographic modeling of the ventral attention network. *J. Neurol. Sci.* 408:116548. 10.1016/j.jns.2019.116548 31707250

[B3] American Psychiatric Association (2013). *Diagnostic and statistical manual of mental disorders : DSM-5, ed. American Psychiatric Assocation and American Psychiatric Association DSM Task Force.* Arlington, VA: American Psychiatric Association.

[B4] Arnulf I. Konofal E. Merino-Andreu M. Houeto J. L. Mesnage V. Welter M. L. (2002). Parkinson’s disease and sleepiness: An integral part of PD. *Neurology* 58 1019–1024. 10.1212/wnl.58.7.1019 11940685

[B5] Behzadi Y. Restom K. Liau J. Liu T. T. (2007). A component based noise correction method (CompCor) for BOLD and perfusion based fMRI. *NeuroImage* 37 90–101. 10.1016/j.neuroimage.2007.04.042 17560126 PMC2214855

[B6] Buysse D. J. Yu L. Moul D. E. Germain A. Stover A. Dodds N. E. (2010). Development and validation of patient-reported outcome measures for sleep disturbance and sleep-related impairments. *Sleep* 33 781–792. 10.1093/sleep/33.6.781 20550019 PMC2880437

[B7] Chen T. Guestrin C. (2016). XGBoost: A scalable tree boosting system. *arXiv* [Preprint] arXiv:1603.02754.

[B8] Christensen L. Mendoza J. L. (1986). A method of assessing change in a single subject: An alteration of the RC index. *Behav. Ther.* 17 305–308.

[B9] Dai X. J. Liu B. X. Ai S. Nie X. Xu Q. Hu J. (2020). Altered inter-hemispheric communication of default-mode and visual networks underlie etiology of primary insomnia : Altered inter-hemispheric communication underlie etiology of insomnia. *Brain Imag. Behav.* 14 1430–1444. 10.1007/s11682-019-00064-0 31011953

[B10] Doyen S. Nicholas P. Poologaindran A. Crawford L. Young I. M. Romero-Garcia R. (2022). Connectivity-based parcellation of normal and anatomically distorted human cerebral cortex. *Hum. Brain Mapp.* 43 1358–1369. 10.1002/hbm.25728 34826179 PMC8837585

[B11] Doyen S. Taylor H. Nicholas P. Crawford L. Young I. Sughrue M. E. (2021). Hollow-tree super: A directional and scalable approach for feature importance in boosted tree models. *PLoS One* 16:e0258658. 10.1371/journal.pone.0258658 34695143 PMC8544862

[B12] Espie C. A. Inglis S. J. Harvey L. (2001). Predicting clinically significant response to cognitive behavior therapy for chronic insomnia in general medical practice: Analysis of outcome data at 12 months posttreatment. *J. Consult. Clin. Psychol.* 69 58–66. 10.1037//0022-006x.69.1.58 11302278

[B13] Fasiello E. Gorgoni M. Scarpelli S. Alfonsi V. Ferini Strambi L. De Gennaro L. (2022). Functional connectivity changes in insomnia disorder: A systematic review. *Sleep Med. Rev.* 61:101569. 10.1016/j.smrv.2021.101569 34902821

[B14] Feng J. Zhang Q. Zhang C. Wen Z. Zhou X. (2019). The Effect of sequential bilateral low-frequency rTMS over dorsolateral prefrontal cortex on serum level of BDNF and GABA in patients with primary insomnia. *Brain Behav.* 9:e01206. 10.1002/brb3.1206 30609300 PMC6379591

[B15] Fortier-Brochu E. Beaulieu-Bonneau S. Ivers H. Morin C. M. (2012). Insomnia and daytime cognitive performance: A meta-analysis. *Sleep Med. Rev.* 16 83–94. 10.1016/j.smrv.2011.03.008 21636297

[B16] Garyfallidis E. Brett M. Amirbekian B. Rokem A. van der Walt S. Descoteaux M. (2014). Dipy, a library for the analysis of diffusion MRI data. *Front. Neuroinform.* 8:8. 10.3389/fninf.2014.00008 24600385 PMC3931231

[B17] Glasser M. F. Coalson T. S. Robinson E. C. Hacker C. D. Harwell J. Yacoub E. (2016). A multi-modal parcellation of human cerebral cortex. *Nature* 536 171–178. 10.1038/nature18933 27437579 PMC4990127

[B18] Goulden N. Khusnulina A. Davis N. J. Bracewell R. M. Bokde A. L. McNulty J. P. (2014). The salience network is responsible for switching between the default mode network and the central executive network: Replication from DCM. *NeuroImage* 99 180–190. 10.1016/j.neuroimage.2014.05.052 24862074

[B19] Gruwez A. Libert W. Ameye L. Bruyneel M. (2017). Reliability of commercially available sleep and activity trackers with manual switch-to-sleep mode activation in free-living healthy individuals. *Int. J. Med. Inform.* 102 87–92. 10.1016/j.ijmedinf.2017.03.008 28495352

[B20] He D. Ren D. Guo Z. Jiang B. (2022). Insomnia disorder diagnosed by resting-state fMRI-based SVM classifier. *Sleep Med.* 95 126–129. 10.1016/j.sleep.2022.04.024 35576773

[B21] Hertrich I. Dietrich S. Ackermann H. (2020). The Margins of the Language Network in the Brain. *Front. Commun.* 5:519955. 10.3389/fcomm.2020.519955

[B22] Hyde M. O’Driscoll D. M. Binette S. Galang C. Tan S. K. Verginis N. (2007). Validation of actigraphy for determining sleep and wake in children with sleep disordered breathing. *J. Sleep Res.* 16 213–216. 10.1111/j.1365-2869.2007.00588.x 17542951

[B23] Jiang B. He D. Guo Z. Mu Q. Zhang L. (2019). Efficacy and placebo response of repetitive transcranial magnetic stimulation for primary insomnia. *Sleep Med.* 63 9–13. 10.1016/j.sleep.2019.05.008 31600660

[B24] Kalmbach D. A. Cheng P. Roth T. Sagong C. Drake C. L. (2020). Objective sleep disturbance is associated with poor response to cognitive and behavioral treatments for insomnia in postmenopausal women. *Sleep Med.* 73 82–92. 10.1016/j.sleep.2020.04.024 32799029 PMC7487035

[B25] Kar S. K. (2019). Predictors of Response to Repetitive Transcranial Magnetic Stimulation in Depression: A Review of Recent Updates. *Clin. Psychopharmacol. Neurosci.* 17 25–33. 10.9758/cpn.2019.17.1.25 30690937 PMC6361049

[B26] Krystal A. D. Edinger J. D. Wohlgemuth W. K. Marsh G. R. (2002). NREM sleep EEG frequency spectral correlates of sleep complaints in primary insomnia subtypes. *Sleep* 25 630–640. 12224842

[B27] Lanza G. Cantone M. Lanuzza B. Pennisi M. Bella R. Pennisi G. (2015). Distinctive patterns of cortical excitability to transcranial magnetic stimulation in obstructive sleep apnea syndrome, restless legs syndrome, insomnia, and sleep deprivation. *Sleep Med. Rev.* 19 39–50. 10.1016/j.smrv.2014.04.001 24849846

[B28] Li G. Zhang X. Zhang J. Wang E. Zhang H. Li Y. (2018). Magnetic resonance study on the brain structure and resting-state brain functional connectivity in primary insomnia patients. *Medicine* 97:e11944. 10.1097/MD.0000000000011944 30142814 PMC6113012

[B29] Lie J. D. Tu K. N. Shen D. D. Wong B. M. (2015). Pharmacological Treatment of Insomnia. *P T* 40 759–771. 26609210 PMC4634348

[B30] Liu X. Zheng J. Liu B. X. Dai X. J. (2018). Altered connection properties of important network hubs may be neural risk factors for individuals with primary insomnia. *Sci. Rep.* 8:5891. 10.1038/s41598-018-23699-3 29651014 PMC5897381

[B31] Ma H. Lin J. He J. Lo D. Tsang H. (2021). Effectiveness of TES and rTMS for the Treatment of Insomnia: Meta-Analysis and Meta-Regression of Randomized Sham-Controlled Trials. *Front. Psychiat.* 12:744475. 10.3389/fpsyt.2021.744475 34744835 PMC8569107

[B32] Ma X. Jiang G. Fu S. Fang J. Wu Y. Liu M. (2018). Enhanced Network Efficiency of Functional Brain Networks in Primary Insomnia Patients. *Front. Psychiat.* 9:46. 10.3389/fpsyt.2018.00046 29515469 PMC5826384

[B33] Morgenthaler T. Alessi C. Friedman L. Owens J. Kapur V. Boehlecke B. (2007). Practice parameters for the use of actigraphy in the assessment of sleep and sleep disorders: An update for 2007. *Sleep* 30 519–529. 10.1093/sleep/30.4.519 17520797

[B34] Morin C. M. Drake C. L. Harvey A. G. Krystal A. D. Manber R. Riemann D. (2015). Insomnia disorder. *Nat. Rev. Dis. Prim.* 1:15026. 10.1038/nrdp.2015.26 27189779

[B35] Omniscient Neurotechnology (2020). *Infinitome.* Available online at: https://o8t.com (accessed November 21, 2021).

[B36] Pang R. Zhan Y. Zhang Y. Guo R. Wang J. Guo X. (2017). Aberrant Functional Connectivity Architecture in Participants with Chronic Insomnia Disorder Accompanying Cognitive Dysfunction: A Whole-Brain, Data-Driven Analysis. *Front. Neurosci.* 11:259. 10.3389/fnins.2017.00259 28553199 PMC5425485

[B37] Rossman J. (2019). Cognitive-Behavioral Therapy for Insomnia: An Effective and Underutilized Treatment for Insomnia. *Am. J. Lifestyle Med.* 13 544–547. 10.1177/1559827619867677 31662718 PMC6796223

[B38] Shi X. Guo Y. Zhu L. Wu W. Hordacre B. Su X. (2021). Electroencephalographic connectivity predicts clinical response to repetitive transcranial magnetic stimulation in patients with insomnia disorder. *Sleep Med.* 88 171–179. 10.1016/j.sleep.2021.10.017 34773788

[B39] Sweetman A. Lechat B. Catcheside P. G. Smith S. Antic N. A. O’Grady A. (2021). Polysomnographic Predictors of Treatment Response to Cognitive Behavioral Therapy for Insomnia in Participants With Co-morbid Insomnia and Sleep Apnea: Secondary Analysis of a Randomized Controlled Trial. *Front. Psychol.* 12:676763. 10.3389/fpsyg.2021.676763 34017296 PMC8129160

[B40] Tagliazucchi E. Laufs H. (2014). Decoding wakefulness levels from typical fMRI resting-state data reveals reliable drifts between wakefulness and sleep. *Neuron* 82 695–708. 10.1016/j.neuron.2014.03.020 24811386

[B41] Tagliazucchi E. von Wegner F. Morzelewski A. Borisov S. Jahnke K. Laufs H. (2012). Automatic sleep staging using fMRI functional connectivity data. *NeuroImage* 63 63–72. 10.1016/j.neuroimage.2012.06.036 22743197

[B42] Tahmasian M. Noori K. Samea F. Zarei M. Spiegelhalder K. Eickhoff S. B. (2018). A lack of consistent brain alterations in insomnia disorder: An activation likelihood estimation meta-analysis. *Sleep Med. Rev.* 42 111–118. 10.1016/j.smrv.2018.07.004 30093361 PMC7965842

[B43] Taylor J. J. Kurt H. G. Anand A. (2021). Resting State Functional Connectivity Biomarkers of Treatment Response in Mood Disorders: A Review. *Front. Psychiat.* 12:565136. 10.3389/fpsyt.2021.565136 33841196 PMC8032870

[B44] Van de Water A. T. Holmes A. Hurley D. A. (2011). Objective measurements of sleep for non-laboratory settings as alternatives to polysomnography–a systematic review. *J. Sleep Res.* 20 183–200. 10.1111/j.1365-2869.2009.00814.x 20374444

[B45] van der Werf Y. D. Altena E. van Dijk K. D. Strijers R. L. De Rijke W. Stam C. J. (2010). Is disturbed intracortical excitability a stable trait of chronic insomnia? A study using transcranial magnetic stimulation before and after multimodal sleep therapy. *Biol. Psychiat.* 68 950–955. 10.1016/j.biopsych.2010.06.028 20728874

[B46] Wei Y. Leerssen J. Wassing R. Stoffers D. Perrier J. Van Someren E. (2020). Reduced dynamic functional connectivity between salience and executive brain networks in insomnia disorder. *J. Sleep Res.* 29:e12953. 10.1111/jsr.12953 32164035 PMC7154624

[B47] Yan C. Q. Wang X. Huo J. W. Zhou P. Li J. L. Wang Z. Y. (2018). Abnormal Global Brain Functional Connectivity in Primary Insomnia Patients: A Resting-State Functional MRI Study. *Front. Neurol.* 9:856. 10.3389/fneur.2018.00856 30450072 PMC6224336

[B48] Zhou F. Zhao Y. Huang M. Zeng X. Wang B. Gong H. (2018). Disrupted interhemispheric functional connectivity in chronic insomnia disorder: A resting-state fMRI study. *Neuropsychiat. Dis. Treat.* 14 1229–1240. 10.2147/NDT.S162325 29795981 PMC5957476

